# Prévalence de l´infertilité masculine dans un hôpital universitaire au Maroc

**DOI:** 10.11604/pamj.2021.38.46.19633

**Published:** 2021-01-15

**Authors:** Mohammed Frikh, Mostafa Benaissa, Jalal Kasouati, Yassine Benlahlou, Omar Chokairi, Malika Barkiyou, Meryama Chadli, Adil Maleb, Mostafa Elouennass

**Affiliations:** 1 Service de Bactériologie, Université Mohammed V, Faculté de Médecine et Pharmacie, Hôpital Militaire d´Instruction Mohammed V, Rabat, Maroc,; 2 Laboratoire d´Histo-embryologie et Cytogénétique, Faculté de Médecine et de Pharmacie, Université Mohamed V, Rabat, Maroc,; 3 Service d´Hygiène et de Médecine de Collectivité, Hôpital Militaire d´Instruction Mohamed V, Université Mohammed V, Rabat, Maroc,; 4 Université Mohammed Premier, Faculté de Médecine Oujda, Oujda, Maroc

**Keywords:** Infertilité masculine, facteurs de risque, spermogramme, azoospermie, Male infertility, risk factors, spermogram, azoospermia

## Abstract

**Introduction:**

l´infertilité du couple est devenue ces dernières années un problème de santé publique, dont l´origine peut être féminine, masculine ou mixte. L´origine masculine est incriminée dans 40% des cas. Au Maroc, la plupart des publications ont été axées sur les étiologies et les facteurs de risque de l´infertilité masculine. L´objectif de notre étude est l´évaluation de la prévalence de l´infertilité masculine et le profil des paramètres spermatiques chez les hommes infertiles ou à risque d´infertilité au sein d´un hôpital tertiaire à Rabat.

**Méthodes:**

analyse de 482 patients adressés pour bilan d´infertilité du couple ou dans le cadre du bilan préopératoire d´une varicocèle ou d´une ectopie testiculaire. Les données démographiques, les facteurs de risque d´infertilité, le caractère primaire ou secondaire de l´infertilité ont été enregistrées pour chaque patient. Les paramètres spermatiques ont été étudiés et interprétés selon les normes actualisées de l´OMS en 2010 avec études des facteurs associés à leur perturbation.

**Résultats:**

l´âge moyen des patients était de 35,35±8,81 ans. L´infertilité était primaire dans 61,8%. Les facteurs de risque d´infertilité les plus fréquemment retrouvés sont le tabac suivi par la varicocèle et l´infection. Le spermogramme était perturbé dans 53,1%. L´anomalie la plus fréquente était la perturbation de la vitalité (36,9%) suivie par la concentration spermatique (29,7%) et la morphologie (29,3%). L´âge était le seul facteur ayant un impact significatif sur le spermogramme (p=0,002). Les anomalies de la mobilité se voient à partir de 31 ans, la vitalité est perturbée à 34 ans, la morphologie à 35 ans et la concentration à 37 ans. L´azoospermie a été retrouvée dans 16,4% essentiellement associée à l´infertilité primaire. L´oligo-asthéno-tératospermie était l´association la plus représentée (26,2%).

**Conclusion:**

l´infertilité masculine est fréquente dans notre contexte, l´âge étant son principal facteur de risque. La mobilité est le paramètre le plus précocement atteint.

## Abstract (en)

**Introduction:**

infertility in couples has become a public health problem in recent years. It can be related to a problem in the male, female or both. Men infertility accounts for 40% of cases. In Morocco, most studies have focused on the causes and risk factors for male infertility. The purpose of our study was to assess the prevalence of male infertility and semen parameters in infertile men or in men at high risk of developing infertility in a tertiary hospital in Rabat.

**Methods:**

we conducted an analysis of 482 patients referred for evaluation of infertility in the couple or as part of preoperative assessment of varicocele or testicular ectopia. Demographic data, risk factors for infertility, primary or secondary infertility were recorded for each patient. Semen parameters were assessed and interpreted according to WHO standards updated in 2010 based on studies of factors associated with their disturbance.

**Results:**

the average age of patients was 35.35±8.81 years. Primary infertility was found in 61.8% of cases. The most common risk factors for infertility were tobacco followed by varicocele and infection. Spermogram was altered in 53.1% of cases. The most common abnormality was sperm vitality issues (36.9%) followed by spermatic concentration (29.7%) and morphology (29.3%). Age was the unique factor which had a significant impact on spermogram (p=0.002). Abnormalities in mobility were detected in patients ≥31 years, sperm vitality issues in patients aged 34 years, abnormalities in morphology in patients aged 35 years and concentration in patients aged 37 years. Azoospermia was found in 16.4% of cases and was mainly associated with primary infertility. Oligo-astheno-teratozoospermia was the most common association (26.2%).

**Conclusion:**

male infertility is common in our context. The major risk factor is age. Mobility is the earliest parameter achieved.

## Introduction

L´infertilité est définie par le retard de conception après un an de rapport non protégé au sein d´un couple [1]. Ce délai est même raccourci à 6 mois en présence d´un âge avancé de la partenaire (>35 ans) ou d´une notion de cure chirurgicale pour cryptorchidie chez l´homme [2,3]. L´infertilité touche 15% des couples [4]. L´origine masculine est présente dans 40% des cas [5]. L´infertilité peut être attribuée à l´homme quand il existe une altération d´un ou de plusieurs paramètres spermatiques à savoir la concentration spermatique et/ou de la mobilité et/ou de la morphologie dans au moins un de 2 prélèvements de sperme effectués à 4 semaines d´intervalle [6]. Les facteurs de risque sont multiples et varient entre des causes anatomiques, hormonales, infectieuses et toxiques. Au Maroc, quelques rares études se sont intéressées au problème d´infertilité du couple de par les facteurs toxiques, anatomiques et moléculaires [7-10]. L´objectif de cette étude est d´évaluer la prévalence de l´infertilité masculine et le profil des anomalies spermatiques chez les hommes infertiles ou à risque d´infertilité au sein d´un hôpital tertiaire à Rabat.

## Méthodes

Étude transversale sur 3 ans s´étalant de janvier 2015 à Mars 2018 au sein de l´unité de spermiologie de l´Hôpital Militaire d´Instruction Mohamed V à Rabat. Nous avons colligé 482 patients adressés au service pour bilan d´infertilité du couple ou pour bilan des patients à risque d´infertilité à savoir le bilan préopératoire d´une varicocèle ou patient présentant une ectopie testiculaire. Une fiche de renseignement a été établie pour chaque patient comprenant les données démographiques, les facteurs de risque d´infertilité tels que le tabac, la hernie inguinale, l´ectopie testiculaire, la varicocèle et les antécédents d´infections urogénitales notamment les infections sexuellement transmissibles et son caractère primaire ou secondaire.

Pour l´ensemble des patients, un recueil du sperme a été réalisé au laboratoire, avec un délai d´abstinence allant de 3 à 5 jours selon les normes du guide de bonne exécution des analyses de biologie médicale (GBEA) (1994) [11], et en dehors de tout syndrome infectieux ou épisode fébrile. L´analyse du sperme a été faite manuellement après un temps de liquéfaction de 30 min à 37°. L´ensemble des caractères physico-chimiques, volume, mobilité (a+b), vitalité (vita -eosine RAL) et concentration spermatique ont été analysés. Les anomalies morphologiques ont été étudiées après coloration des frottis par la technique de Schoor et interprétés selon David [12](1975) et modifiée par Auger et Eustache (2000) [13]. L´ensemble des paramètres spermatiques ont été étudiés et interprétés selon les normes actualisées de l´OMS en 2010 [1].

**L´analyse statistique**: a été réalisée par le logiciel SPSS version 2013. Les variables quantitatives (âge) ont été exprimées en moyenne ± écart-type et les variables qualitatives en pourcentage et effectifs. La relation entre les facteurs de risque d´infertilité et les anomalies du spermogramme a été faite par le test Chi^2^. Pour évaluer la corrélation entre l´âge et les paramètres spermatiques, nous avons utilisé le test de corrélation de Pearson. Les résultats ont été considérés significatifs pour un p <0.05.

**Ethique**: tous les patients ont signé un consentement écrit sur l´utilisation de leurs données à des fins scientifiques.

## Résultats

L´âge moyen des patients est de 35,35±8,81 avec des extrêmes de 16 et 65. L´infertilité était primaire dans 61,8%. Les facteurs de risque d´infertilité les plus fréquemment retrouvés sont le tabac suivi par la varicocèle et l´infection. Le spermogramme était perturbé dans 53,1%. L´anomalie la plus fréquente est la perturbation de la vitalité (36,9%) suivie par la concentration spermatique (29,7%) et le spermocytogramme (29,3%) (Tableau 1). L´analyse des différents facteurs de risque étudiés (Tableau 2) ne trouve pas d´impact significatif sur le spermogramme en dehors de l´âge (p=0,002). Chez les patients ayant un spermogramme anormal, 26,2% avaient des antécédents de varicocèle (p=0,89), 26,6% étaient tabagiques (p=0,28), 23% avaient des antécédents (ATCD) d´infections urogénitales (p=0,72), seuls 6,6% avaient des ATCD d´hernie inguinale ou d´ectopie testiculaire (p=0,19) et 53,9% étaient âgés de plus de 35 ans (p=0,002) (Tableau 3). En effet, il existe une corrélation négative, quoique faible, entre l´âge et les paramètres spermatiques: volume (r=-0,16, p=0,036), numération (r=0,10, p=0,013), mobilité (r=-0,25, p=0,0001), vitalité (r=-0,21, p=0.0001), cytogramme (r=-0,16, p=0,0001) et quelque soit le paramètre étudié, les anomalies sont retrouvées de façon significative pour un âge ≥36 ans (Tableau 4). Le Tableau 4 détermine l´âge d´apparition des différentes anomalies du spermogramme. En effet, le premier paramètre à être perturbé est la mobilité à partir de 31 ans, la vitalité à partir de 34 ans, le spermocytogramme à 35 ans et ce n´est qu´à partir de 37 ans que la concentration diminue; le volume du sperme étant le dernier à être perturbé à 38 ans. Les anomalies les plus fréquentes sont la tératospermie (41%) et l´oligospermie (40,2%) suivis par l´asthénospermie (29,7%). L´azoospermie a été retrouvée dans 16,4%. Les associations d´anomalies sont représentées essentiellement par l´oligo-asthéno-tératospermie (OATS) avec 26,2% (Tableau 5). Les facteurs associés à l´azoospermie sont le type d´infertilité (p 0,02). L´azoospermie est plus retrouvée dans l´infertilité primaire (83,3%) (Tableau 6), on note par contre juste une tendance d´association de l´âge et des ATCD d´infection à l´azoospermie (P=0,07). Concernant l´OATS, nous n´avons trouvé aucun facteur associé de façon significative.

**Tableau 1:** données démographiques et paramètres spermatiques de la population étudiée

Variables (N 482)	N(%)
**Age moyen**	35.35 ± 8.81*
< 36 ans	252(52.3)
≥ 36 ans	228(47.3)
**Infertilité**	
Célibataire	68(14.1)
Primaire	298(61.8)
Secondaire	116(24.1)
**FDR d’infertilité**	
Tabac	138(28.6)
HI/ET	26(5.4)
Varicocèle	125(25.9)
Infection	108(22.4)
**Paramètres spermatiques**	
Volume spermatique < 1.5 ml	37(7.7)
Concentration spermatique < 15M/ml	143(29.7)
Mobilité (a+b) <32%	106(22)
Vitalité <58%	178(36.9)
Spermocytogramme<15%	141(29.3)
Spermogramme anormal	256(53.1)

* Moyenne ± écart-type, HI: hernie inguinal, ET: ectopie testiculaire, a+b: mobilité rapide et progressive

**Tableau 2:** prévalence des facteurs de risque chez les patients ayant une perturbation du spermogramme

Variables	N	Présence du FDR	Absence du FDR	p
**Age ≥ 36 ans**	256	138(53.9)	118(46.1)	**0.002**
**Tabac**	138	68(26.6)	188(73.8)	0.28
**HI+ET**	26	17(6.6)	239(93.4)	0.19
**Varicocèle**	125	67(26.2)	189(73.8)	0.89
**Infection**	108	59(23.0)	197(77.0)	0.72

**FDR:** facteur de risque, **HI:** hernie inguinal, **ET:** ectopie testiculaire

**Tableau 3:** influence de l´âge sur les paramètres spermatiques

Anomalies PS	N	Age <36	Age ≥36	P
**Volume**	37	13(35.1)	24(64.9)	**0.039**
**Numération**	143	64(44.8)	79(55.2)	**0.02**
**Mobilité**	106	34(32.1)	72(67.9)	**0.0001**
**Vitalité**	178	78(43.8)	100(56.2)	**0.003**
**Cytologie**	141	60(42.6)	81(57.4)	**0.004**
**Azoospermie**	42	14(33.3)	28(66.7)	**0.009**

PS: paramètres spermatiques

**Tableau 4:** âge de survenue des perturbations des paramètres spermatiques

	38 ans	37 ans	36 ans	35 ans	34 ans	33 ans	32 ans	31 ans	30 ans
**Volume**	**0.03**	0.054	0.08	0.21	0.20	0.5			
**Concentration**	**0.002**	**0.01**	0.056	0.07	0.18	0.52			
**Mobilité**	**0.0001**	**0.0001**	**0.0001**	**0.0001**	**0.0001**	**0.004**	**0.001**	**0.01**	0.06
**Vitalité**	**0.01**	**0.007**	**0.002**	**0.01**	**0.048**	0.1			
**SCG**	**0.01**	**0.01**	**0.01**	**0.02**	0.05	0.34			


SCG: spermocytogramme, résultats exprimés en p calculé par le test khi 2 en comparant le paramètre étudié chez les sujets de moins de « l´âge cité sur la colonne » avec les sujets dont l´âge est supérieur ou égal à « l´âge cité sur la colonne ».

**Tableau 5:** prévalence des différents types d’anomalie du spermogramme

Type d’anomalie	Nombre (N)	Pourcentage (%)
**Azoospermie**	42	**16.4**
**Asthénospermie**	76	**29.7**
**Anomalie de volume (hypospermie ou hyperspermie)**	20	7.8
**Oligospermie**	103	**40.2**
**Nécrozoospermie**	67	**26**.2
**Tératospermie**	105	**41.0**
**OATS**	67	26.2
**Asthéno-nécro**	5	2
**Oligo-necro**	4	1.6
**Térato-nécro**	10	3.9

OATS: oligo-asthéno-tératospermie

**Tableau 6:** facteurs associés à l´azoospermie et à l’OAT

Facteurs associés	Azoospermie			OATS		
	N	%	p	N	%	p
**Age <36 ans Age ≥36 ans**	14 28	33.3 66.7	**0.07**	27 40	40.3 59.7	0.16
**Infection**	14	33.3	**0.07**	17	25.4	0.35
**Tabac**	11	26.2	0.95	19	28.4	0.40
**Hernie inguinale et l´ectopie testiculaire**	4	9.5	0.41	4	6.0	0.52
**Varicocèle**	10	23.8	0.74	21	31.3	0.16
**Infertilité**						
**Primaire**	35	83.3	**0.02**	41	61.2	0.29
**Secondaire**	4	9.5		19	28.4	
**Célibataire**	3	7.1		7	10.4	

**OATS**: oligo-asthéno-tératospermie; **N**=nombre; **%**= pourcentage

## Discussion

**L´âge moyen des patients qui consultent pour stérilité**: l´âge moyen de nos patients est de 35.35 ans alors que dans la littérature, il varie entre 37 et 39 ans [8,14-16].Cet âge jeune peut être expliqué par le mode de recrutement de nos patients qui ne se limite pas aux consultants pour bilan d´infertilité mais aussi les célibataires qui présentent un risque d´infertilité. Après avoir exclu cette dernière catégorie, l´âge moyen est de 37,92±8,14 ce qui rejoint la plupart des séries sus cités. Cet âge avancé reflète d´une part l´âge avancé du mariage dans notre population estimé à 31,3 ans chez les hommes [17] mais également au barrage psychologique des hommes par rapport à leur infertilité et donc à la consultation. De plus les contraintes sociales et professionnelles actuelles ont conduit à un changement du style de vie des populations et un retard du désir de procréation [18].

**Primaire/secondaire**: nous avons enregistré près de 62% d´infertilité primaire. Ce taux est variable selon les séries et se situe entre 57,4 et 82% [8,9,15,16]. Ceci peut être dû à la prévalence variable des facteurs d´infertilité secondaire dans les différents pays avec notamment l´âge qui diminue l´index de fécondabilité chez les 2 sexes et l´altération des paramètres spermatiques chez l´homme avec l´âge [19]. Un autre facteur est le style de vie avec notamment l´effet délétère de la cigarette qui peut expliquer l´infertilité masculine en absence d´altération des paramètres spermatiques; le tabac pouvant engendrer des anomalies chromosomiques ce qui explique que bien que ce dernier était le 2^e^ facteur de risque retrouvé chez nos patients, l´effet sur le spermogramme n´était pas significatif. Par ailleurs, dans l´étude de Benksim *et al*. 2018, les principales causes d´infertilité primaire sont entièrement dues à des causes masculines alors que les causes d´infertilité secondaire sont majoritairement féminines [9].

**Responsabilité masculine dans l´infertilité du couple**: selon l´étude d´Agarwal *et al*. 2015, le pourcentage des hommes infertiles varie entre 2,5 et 12%. Ce taux serait plus élevé en Afrique et en Europe central et de l´Est [20]. L´origine masculine de l´infertilité varie selon les séries: 8% selon l´OMS (1993) à travers une étude de 8500 couples infertiles et 35% de causes combinées [21]. Mais selon une méta-analyse plus récente, les hommes sont seuls en cause dans 20-30% et contribuent à 50% de tous les cas d´infertilité [20]. Au Maroc, à travers une série portant sur 1265 couples infertiles, l´origine masculine a été enregistrée dans 45,2% [8]. Dans notre série, elle est de 53,1% selon les résultats du spermogramme. Elle augmente à 53,6% après exclusion des patients célibataires. L´analyse des causes féminines n´étant pas explorée dans notre étude, nos résultats reflèteraient la contribution masculine à tous les cas d´infertilité. Ce qui rejoint les données de la littérature.

**Les facteurs de risque incriminés et leur influence**: le taux élevé d´infertilité masculine a conduit Levine *et al*. (2017) à analyser l´évolution de la qualité du sperme, à travers une méta-analyse de 185 études de sujets masculins sans problème d´infertilité [22]. Les résultats de l´étude ont montré que le taux des spermatozoïdes a diminué de 50-60% entre 1973 et 2011 dans les pays industrialisés ce qui soulève l´hypothèse de facteurs environnementaux dans la dégradation de la qualité du sperme avec en particulier le régime alimentaire [23], l´obésité et les toxiques industriels [24-26]. Dans notre étude, nous avons analysé les facteurs de risque les plus importants avec en particulier l´âge, la varicocèle, les infections urogénitales, les antécédents d´hernie inguinale ou d´ectopie testiculaire, mais également les facteurs toxiques avec essentiellement le tabac.

**La varicocèle**: est considérée comme la première cause d´infertilité masculine avec une prévalence pouvant atteindre 40% des hommes infertiles [27]. La varicocèle peut altérer la spermatogénèse selon différents mécanismes [28,29]. La cure chirurgicale bien qu´elle améliore la numération et la mobilité et de ce fait la fertilité naturelle ou les techniques de reproduction assistée [30], elle ne corrige pas le taux des anomalies morphologiques [31]. L´antécédent de varicocèle a été retrouvé chez 25% de nos patients dont plus de la moitié (53%) avait un spermogramme anormal sans toutefois de corrélation significative. Alors que les patients avec infertilité biologique confirmé par le spermogramme, 26% avait une varicocèle ce qui rejoint les données de l´OMS (1992) [32].

**L´infection**: a été enregistrée chez 22,4% de nos patients, ce qui rejoint les taux rapportés dans la littérature 8-35% [33]. En effet d´une part, il existe une élévation de la charge bactérienne séminale chez les patients hypofertiles [34]. Et d´autres part, environ 10% des hommes ayant des antécédents d´épididymite développent plus tard une azoospermie et 30% une oligozoospermie. De même, l´infection peut entrainer une obstruction des canaux excréteurs et des anomalies de la spermatogénèse post infectieuse [35]. Différents germes sont impliqués avec en particulier *Chlamydia trachomatis, Ureaplasma urealyticum* et *Mycoplasma genitalium, Mycoplasma hominis, Neisseria gonorrhoeae, Gardnerella, Trichomonas vaginalis, Staphylococcus aureus, Staphylococcus coagulase négative, Streptococcus, Corynebacterium, E. coli* [33,36-39]. Récemment, Benbella *et al*. (2017) a rapporté aussi la tuberculose comme infection à risque d´infertilité [8]. De même, Liu *et al*. (2013) a ajouté le virus de l´hépatite B aux germes qui perturbent la qualité du sperme et ceci par un phénomène auto-immun [40].

**La hernie inguinale et l´ectopie testiculaire** ont été enregistrées dans 5,4% des patients. En effet, 90% des patients ayant une cryptorchidie bilatérale non traités développent une azoospermie. Dans les autres cas, à savoir cryptorchidie unilatérale, ectopique, inguinale, traité ou non traitée, la fertilité et la possibilité de paternité reste imprédictible [41]. La hernie inguinale est incriminée dans l´infertilité après la cure chirurgicale qui peut être à l´origine d´une azoospermie excrétoire par obstruction iatrogène des canaux déférents qui traversent le canal inguinal [42]. En effet, leur prévalence est faible dans notre série et sans corrélation significative avec les anomalies du spermogramme.

**Le tabac**: est le facteur de risque le plus retrouvé dans notre série (28%). Dans une étude de prévalence des facteurs de risque d´infertilité du couple, le tabac a été enregistré chez 34% des hommes infertiles. Il est considéré comme le facteur le plus nocif avec l´alcool sur la fertilité. Son effet délétère passe par la perturbation des taux hormonaux par la nicotine et les autres produits chimiques et de ce fait altère les paramètres spermatiques [43,44] mais aussi par les dommages causés à l´ADN de la lignée germinale via le stress oxydatif [45,46].

**D´autres facteurs** de risque non explorés dans notre étude ont été rapportés avec notamment l´obésité qui est associée à une concentration spermatique plus faible [47] et à une moins bonne réponse au traitement de l´infertilité [3,48]. En outre, l´obésité paternelle affecte le phénotype métabolique et reproductif de la descendance par une reprogrammation épigénétique des cellules souches spermatogoniales [49]. D´autres facteurs alimentaires [50] et toxiques diminuent la numération et altèrent la morphologie des spermatozoïdes tels que le soja, les acides gras saturés et les acides gras « trans » [47,51,52], les viandes transformées [53,54] et les fruits et légumes à haut résidu de pesticides [55].

**Les perturbations des paramètres spermatiques**: les paramètres spermatiques dans notre série étaient altérés avec des degrés variables et notamment une plus grande fréquence des anomalies de la vitalité suivies par la concentration spermatique et la morphologie et à un moindre degré la mobilité. Les modalités d´altération des paramètres spermatiques dépendent des facteurs de risque de la population étudiée. Dans notre série, seul l´âge était significativement associé à l´altération de l´ensemble des paramètres. En effet, plusieurs auteurs rapportent que l´âge avancé est associé à un déclin du volume séminal, de la mobilité et la morphologie spermatique mais pas de la concentration spermatique [56-58]. Dans la série de Kuman, l´âge déterminant était de 35 ans à partir duquel la numération, la mobilité et la morphologie sont diminuées. Ce qui rejoint les données de notre série, avec notamment l´altération probable de 3 paramètres à partir de 35 ans mais différents de ceux de Kuman avec en particulier la mobilité, la vitalité et la morphologie alors que la numération est atteinte tardivement à 37 ans. Par contre, la mobilité serait atteinte précocement à partir de 31 ans puis la vitalité à 34 ans ce qui peut supposer que d´autres facteurs associés, non explorés dans notre étude, interviendraient dans ce profil d´altérations des paramètres spermatiques. D´autre part, l´altération précoce de la mobilité et de la vitalité rendrait compte de l´augmentation du taux d´infertilité sous nos cieux. L´âge du mariage se situant à 31.3 ans, l´infertilité primaire s´en trouve augmentée. Par ailleurs, l´âge paternel avancé est également associé à une augmentation de la prévalence des anomalies génomiques et épigénomique du sperme [59] ce qui peut expliquer l´infertilité chez des patients n´ayant pas d´altération des paramètres spermatiques et le taux d´échec des mesures d´aide à la procréation chez des patients en âge avancé [60,61]. L´association des anomalies des paramètres spermatiques a été enregistrée chez 33.6% des patients avec en particulier l´OATS dans plus d´un quart des patients. Selon Pontonnier *et al*. 1993, cette dernière constitue l´anomalie la plus fréquente dans la population générale [62]. Elle intéresserait jusqu´à 30% des hommes infertiles [63]. Elle serait liée à l´âge qui diminue progressivement le volume spermatique et la mobilité des spermatozoïdes [64], à l´épididymite par le biais d´une altération de la méthylation de l´ADN des gamètes [65], et aux infections asymptomatiques liées à l’herpès, aux adénovirus et à *Chlamydia trachomatis* [66,67]. Dans notre série, aucun facteur n´a été associé de façon significative à l´OATS ce qui suggère le dernier mécanisme cité à savoir l´infection asymptomatique. En effet, dans une autre étude de l´équipe [68], la prévalence de l´infection à *Chlamydia trachomatis* était de 58,5% chez les patients avec spermogramme anormal.

L´azoospermie a été retrouvée dans 16,4% des cas. Sa prévalence est estimée à 10 à 15% des hommes infertiles [69]. Elle résulte de causes pré-testiculaires, testiculaires et post testiculaires. Elle est classée en causes obstructives (40%) et non obstructives (AUO 2010) [70]. Les causes obstructives comprennent: les anomalies congénitales ou post infectieuses des voies génitales notamment la tuberculose dans notre contexte [71], les obstructions iatrogènes après cure chirurgicale d´une hernie inguinale [42,72] ou d´une cryptorchidie [73]. Les causes non obstructives incluent les antécédents de cryptorchidie, de chimiothérapie, d´exposition à des radiations environnementales et de traitement par testostérone [74] mais aussi d´infection notamment à Chlamydia [36,37] et à HIV [75]. Les causes génétiques représentent 10-15% des causes non obstructives [76]. Dans notre série, l´azoospermie a été associée à l´infertilité primaire (P=0,02) avec une tendance à l´association avec les antécédents d´infection et l´âge avancé des patients ce qui peut plaider pour une origine génétique, hormonale ou encore infectieuse, aucune association n´ayant été enregistrée avec la varicocèle, la hernie inguinale ou l´ectopie testiculaire. Les limites de notre étude résident dans la non-exploration de tous les facteurs de risque notamment génétiques et hormonaux et la non-implication des facteurs féminins. Toutefois, elle a permis d´identifier une prévalence encore méconnue de l´infertilité masculine au Maroc et de déterminer un profil d´altération des paramètres spermatiques et leur âge de survenue.

## Conclusion

La prévalence de l´infertilité masculine dans notre série est importante, avec une prévalence de 53% dans notre série et une prédominance de l´infertilité primaire. L´âge est le principal facteur de risque associé à l´altération des paramètres spermatiques. La mobilité est atteinte à un âge précoce bien avant l´âge de mariage des hommes dans notre pays ce qui confronte d´emblée la majorité des couples à une infertilité masculine liée à l´âge en dehors d´autres facteurs de risque souvent intriqués.

### Etat des connaissances sur le sujet

- L´infertilité du couple et en particulier l´infertilité masculine est en augmentation partout dans le monde ce qui en fait un sujet d´actualité scientifique;
- Beaucoup de publications s´intéressent à ce sujet mais il y a toujours un manque de données concernant la région du Maghreb et dans les différentes méta-analyses, on trouve souvent une extrapolation des conclusions et des résultats pour notre pays.

### Contribution de notre étude à la connaissance

- Nous avons défini la prévalence de l´infertilité masculine à 53% dans notre étude;
- Nous avons déterminé le profil des anomalies des paramètres spermatiques chez les hommes infertiles; la tératospermie et l´oligospermie étant les plus fréquentes suivies par l´asthénospermie et la nécrozoospermie.
